# Golgi Oncoprotein *GOLPH3* Gene Expression Is Regulated by Functional E2F and CREB/ATF Promoter Elements

**DOI:** 10.3390/genes10030247

**Published:** 2019-03-25

**Authors:** Beatriz Peñalver-González, Jon Vallejo-Rodríguez, Gartze Mentxaka, Asier Fullaondo, Ainhoa Iglesias-Ara, Seth J. Field, Ana M. Zubiaga

**Affiliations:** 1 Department of Genetics, Physical Anthropology and Animal Physiology, University of the Basque Country UPV/EHU, 48080 Bilbao, Spain; beatriz.penalver@ehu.eus (B.P.-G.); jon.vallejo@ehu.eus (J.V.-R.); gartze.mentxaka@ehu.eus (G.M.); asier.fullaondo@ehu.eus (A.F.); ainhoa.iglesias@ehu.eus (A.I.-A.); 2 Division of Endocrinology and Metabolism, University of California, San Diego, La Jolla, CA 92093, USA; sjfield@ucsd.edu

**Keywords:** cell cycle, E2F factors, Golgi, *GOLPH3*, CREB/ATF, gene regulation

## Abstract

The Golgi organelle duplicates its protein and lipid content to segregate evenly between two daughter cells after mitosis. However, how Golgi biogenesis is regulated during interphase remains largely unknown. Here we show that messenger RNA (mRNA) expression of *GOLPH3* and *GOLGA2*, two genes encoding Golgi proteins, is induced specifically in G1 phase, suggesting a link between cell cycle regulation and Golgi growth. We have examined the role of E2F transcription factors, critical regulators of G1 to S progression of the cell cycle, in the expression of Golgi proteins during interphase. We show that promoter activity for *GOLPH3*, a Golgi protein that is also oncogenic, is induced by E2F1-3 and repressed by E2F7. Mutation of the E2F motifs present in the *GOLPH3* promoter region abrogates E2F1-mediated induction of a *GOLPH3* luciferase reporter construct. Furthermore, we identify a critical CREB/ATF element in the *GOLPH3* promoter that is required for its steady state and ATF2-induced expression. Interestingly, depletion of *GOLPH3* with small interfering RNA (siRNA) delays the G1 to S transition in synchronized U2OS cells. Taken together, our results reveal a link between cell cycle regulation and Golgi function, and suggest that E2F-mediated regulation of Golgi genes is required for the timely progression of the cell cycle.

## 1. Introduction

The Golgi complex is an essential organelle in the secretory pathway, in which newly synthesized proteins and lipids undergo posttranslational modifications, and are subsequently sorted to their target locations. The Golgi complex consists of a stack of densely packed and flattened cisternal membranes. Stacks are commonly interconnected and form a ribbon-like structure that resides close to the nucleus. A large body of evidence has shown that the Golgi apparatus is dynamic and is able to disassemble and reassemble under a variety of stress conditions or during cell division cycles through mechanisms that are not well understood [1,2,3].

As the cell progresses through the interphase of the cell cycle, the Golgi apparatus, like other organelles, must duplicate in content and in size before its correct partitioning into daughter cells [1,4]. This process appears to be tightly coupled to cell growth, and probably involves a complex interplay between extracellular signals and intracellular transcriptional and posttranscriptional networks that induce expression of genes encoding organellar proteins. However, how this process occurs and is regulated remains largely unknown.

A detailed cell synchronization analysis at various cell cycle phases has shown that continuous Golgi growth and cell size growth are initiated at late G1 to reach a near doubling at the end of G2 phase [4], coinciding with increasing protein translation of some Golgi components, such as the Golgi structural protein *GOLGA2* (GM130) and the Golgi enzyme MAN2A2 (MannII). The kinase S6K1, whose levels are increased at late G1 phase, is thought to mediate a rise in Golgi protein content [4]. In addition to translational control of Golgi-specific protein production, there is some evidence for transcriptional regulation [5,6].

Major regulation of timely gene expression during G1-to-S progression is carried out by the E2F family of transcription factors (E2F1-8). According to the currently accepted model of cell cycle regulation, in quiescent cells, the transcription of genes required for cell cycle entry and progression is repressed by complexes formed by hypophosphorylated Retinoblastoma-family (Rb) of proteins bound to E2F factors [7], together with a large number of chromatin modifying components. The repressor complexes, which can be formed by different members of the E2F family [8,9], are disrupted upon phosphorylation of Rb pocket proteins by Cyclin/CDK complexes upon entry into the G1 phase. Consequently, free E2F is able to induce the expression of genes necessary for DNA replication and cell cycle progression [10]. Whether E2Fs are involved in regulating the expression of components of cellular organelles such as those residing in the Golgi complex remains to be determined. Given the timely regulation of Golgi growth during the cell cycle, we analyzed the impact of cell cycle regulatory mechanisms on the expression of genes encoding Golgi-specific proteins. We find that *GOLPH3* expression is regulated by E2F factors through E2F motifs present in its promoter. Moreover, we identify a critical CREB/ATF element in the *GOLPH3* promoter that is required for its steady state as well as ATF-induced expression. Our findings uncover a transcriptional regulation of genes encoding Golgi-specific proteins, and present evidence suggesting that the coordinated action of nuclear and Golgi components is necessary for the timely progression of the cell cycle.

## 2. Materials and Methods

### 2.1. Cell Culture and Flow Cytometry

Human U2OS osteosarcoma and mouse NIH 3T3 fibroblast cell lines were maintained in Dulbecco’s modified Eagle’s medium supplemented with fetal bovine serum (10%). For cell synchronization at mitosis, U2OS cell cultures were incubated with thymidine (2 nM) for 20 h. Subsequently, cells were washed and cultured for an additional 15 h in fresh medium. Nocodazole (50 ng/mL) was added to the cultures for the last 10 h. Mitotic cells were collected by shaking off the plates and seeded in complete medium for subsequent analyses. For cell synchronization at G0, NIH3T3 cells were incubated for 48 h with Dubelcco’s modified Eagle’s medium supplemented with 0.1% fetal bovine serum. After G0 synchronization, cells were collected at different times for subsequent analysis.

To assess cell-cycle distribution, cells were fixed with chilled 70% ethanol, stained with 50 µg/mL propidium iodide (PI) and analyzed by flow cytometry (FACSCalibur, BD). The analyses of generated data were performed with Summit 4.3 software.

### 2.2. Plasmid Description

Mammalian expression plasmids pRc-CMV-HA-E2F1, pRc-CMV-HA-E2F2, pRc-CMV-HA-E2F3 have been previously described [11]. Plasmid pCMV6-XL5-E2F7 was purchased from Origene. Plasmids pATF2-HA and pΔ-ATF2-HA were kindly provided by Dr. Lazo-Zbikowski. To construct the wild-type pGL2-*GOLPH3*Promoter-luc (pGP3-WT-luc) reporter plasmid, 488 bp (−254 to +234) of the human *GOLPH3* promoter region was amplified by PCR using human genomic DNA as template (See Supplementary Table S1 for primer sequence information). PCR product was digested with MluI and HindIII and cloned into the pGL2-basic luciferase reporter vector (Promega).

DNA fragments encoding *GOLPH3* promoter deletion mutants lacking the upstream region (pGP3-ΔUR-luc), the downstream region (pGL2-GP3-ΔDR-luc) or containing only a minimal region (pGP3-MR-luc) were amplified by PCR using the pGP3-WT-luc vector as template. PCR products were digested with MluI and HindIII and ligated into pGL2-basic vector to generate the corresponding pGP3-WT deletion mutant.

Site-directed mutagenesis of the CREB/ATF motifs in pGP3-WT-luc reporter plasmid was carried out using the QuickChange Lightning Site-Directed Mutagenesis Kit (Agilent Technologies), following the manufacturer’s directions. Briefly, forward and reverse mutagenic primers containing mutated nucleotides (See Supplementary Table S2 for nucleotide sequences of the primer sets used for mutagenesis) were used to PCR amplify pGP3-WT-luc plasmid. Subsequently, parental DNA digested with *DpnI* and remaining plasmid was transformed into competent cells for nick repair. The resulting plasmid was named pGP3-CREBm-luc.

Site-directed mutagenesis of the two E2F motifs in pGP3-WT-luc reporter plasmid was carried out using the QuickChange Multi Site-Directed Mutagenesis Kit (Agilent Technologies), following the manufacturer’s directions. Briefly, two mutagenic forward primers were used to synthesize the mutant strand using pGP3-WT-luc as template. After *DpnI* digestion of template strand, mutant ssDNA strand was transformed into XL10 Gold competent cells. The resulting plasmid was named pGP3-E2Fm-luc.

Site-directed mutagenesis of the triple mutant for E2F and CREB/ATF motifs was carried out using pGP3-E2Fm-luc as template and CREB forward and reverse mutagenic primers, as indicated above. The resulting plasmid was named pGP3-3xm-luc.

The orientation and integrity of all constructions were confirmed by DNA sequencing.

### 2.3. Transfections and siRNA-Mediated Knockdown

Plasmid transfection was performed with various amounts of DNA in 6-well or 12-well culture dishes using XtremeGENE HD (Roche Pharma, Basel, Switzerland) transfection reagent following manufacturer’s recommendations. The mixture was incubated for 25 min at room temperature and added dropwise to cell cultures.

For knockdown of endogenous *GOLPH3* [12], commercial small interfering RNA (siRNA) oligonucleotides (Life Technologies, Carlsbad, CA, USA) were transfected at a final concentration of 7.5 nM using Lipofectamine RNAiMAX (Life Technologies) following the manufacturer’s recommendation.

### 2.4. Luciferase Activity Assay

For luciferase activity assays in asynchronous cells, U2OS cells were plated at a density of 2 × 10^5^ cells/well on a six-well plate, and 24 h later, cotransfected with 500 ng of the firefly luciferase reporter vector, 20 ng of the *Renilla* luciferase reporter vector (pRL-TK) and varying amounts of expression plasmids. The total amount of DNA in the reaction mixtures was brought to 1 µg using pCMV vector. Using the Dual-Luciferase Reporter Assay System (Promega, Madison, WI, USA), the reporter firefly luciferase activity was measured 24 h or 48 h after transfection, following the manufacturer’s recommendations. Data were normalized to the transfection efficiency estimated by the activity of *Renilla* luciferase in each sample, obtaining Relative Luciferase Units (RLU). The luciferase activity measured in cells transfected only with reporter vector was used as a reference to calculate fold induction.

For luciferase activity assays with synchronized U2OS cells, the transfections were carried out after washing cells following incubation with thymidine (2 nM) for 20 h. Subsequently, cells were cultured for an additional 15 h in fresh medium. Nocodazole (50 ng/mL) was added to the cultures for the last 10 h. Mitotic cells were collected by shaking off the plates and cultured in complete medium for subsequent luciferase assays.

### 2.5. RNA and Protein Expression Analyses

Total RNA extraction was performed with TRIzol Reagent (Life Technologies) and purified using the RNeasy Mini kit (Qiagen, Hilden, Germany) following the manufacturer’s recommendations. RNA was reverse-transcribed into cDNA with the High-Capacity cDNA RT Kit (Life Technologies) and qPCR was performed as described previously [13], following Minimal Information for Publication of Quantitative Real-Time PCR Experiments (MIQE) guidelines. Briefly, qPCR was performed on several cDNA dilutions plus 1 × SYBR green PCR Master Mix (Applied Biosystems, Foster City, CA, USA) and 300–900 nM of primers for the analyzed genes (sequences are listed in Supplementary Table S3). Reactions were carried out in triplicate using QuantStudio 3 (Applied Biosystems) thermocycler for 40 cycles (95 °C for 15 s and 60 °C for 1 min) after an initial 10-min incubation at 95 °C. Relative amounts of cDNA were normalized to the internal control *EIF2C2*, whose levels were invariant in all the analyzed conditions.

For western blot analyses, cells were lysed in buffer containing 10 mM NaPO4 H, pH 7.2; 1 mM EDTA; 1 mM EGTA; 150 mM NaCl; 1% NP-40 and a cocktail of protease and phosphatase inhibitors (Roche). Protein concentrations in supernatants were determined using a commercially available kit (DC Protein Assay from Bio-Rad, Hercules, CA, USA). Twenty to thirty micrograms of protein were loaded per lane, fractionated in 10–12% SDS-PAGE gels and transferred onto nitrocellulose membranes (Bio-Rad), as described previously [13]. Antibodies against the following proteins were used: E2F1 (sc-193, Santa Cruz), E2F2 (sc-633, Santa Cruz, Dallas, TX, USA), E2F3 (sc-878, Santa Cruz), *GOLPH3* (ab91492, Abcam, Cambridge, UK), α-Tubulin TUBA4A (T-9026, Sigma, Saint Louis, MO, USA). Immunocomplexes were visualized with horseradish peroxidase-conjugated anti-rabbit IgG antibodies (Santa Cruz), followed by chemiluminiscence detection (ECL, Amersham) with a ChemiDoc camera (Bio-Rad).

### 2.6. Bioinformatic Tools

Search for transcription factors motifs in *GOLPH3* gene promoter was carried out with the *ConSite* web-based tool for finding *cis*-regulatory elements in genomic sequences (http://consite.genereg.net/). The search was restricted to the proximal promoter region (−500 and +250 relative to the transcriptional star site). A cutoff of 0.80 was applied.

### 2.7. Statistical Analysis

Data are presented as mean ± standard deviation (SD). The significance of the difference between two groups was assessed using the Student two-tailed *t*-test. *p* < 0.05 was considered statistically significant.

## 3. Results

### 3.1. Cell Cycle Regulation of Golgi-Specific Gene Expression

**Figure 1:**
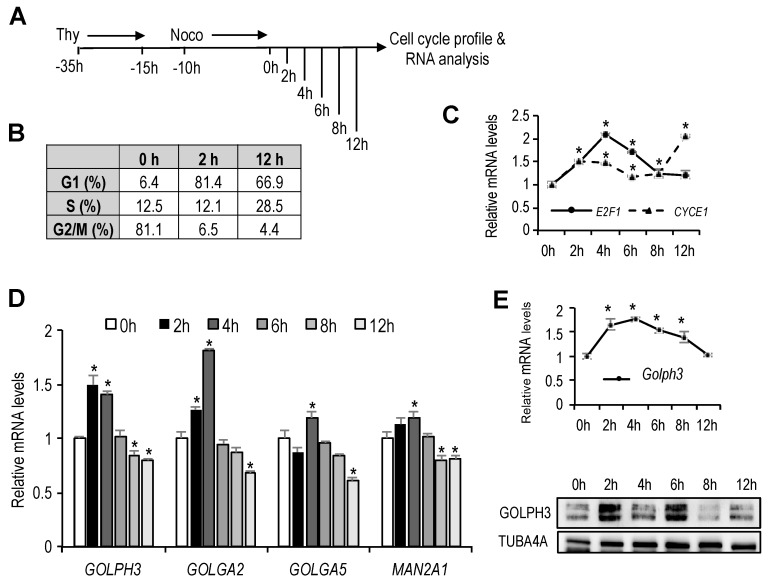
Expression of genes encoding Golgi-specific proteins in the cell cycle. (**A**) As shown in the schematic diagram, U2OS cells that had been treated with thymidine (Thy) for 20 h were washed and 5 h later were treated with nocodazole (Noco) for 10 h to synchronize in mitosis. Subsequently, cells were released into the cell cycle and collected at the indicated time-points for cell cycle profiling and RNA analysis. (**B**) Table showing the percentage of cells in each cell cycle phase at the indicated time-points, as assessed by FACS analysis of PI-stained cells. (**C**,**D**) Reverse transcriptase-quantitative PCR (RT-qPCR) analyses of indicated genes is shown. Expression values were normalized to the expression of *EIF2C2*, used as standard control. Data are represented as fold-change (mean + SD) relative to samples synchronized in mitosis (0 h). Shown are the results of one representative experiment of three independent experiments. (**E**) RT-qPCR analysis of mouse *GOLPH3* mRNA. Mouse NIH 3T3 cells that had been synchronized in G0 by serum starvation were released into the cell cycle after serum addition. RNA samples were collected at the indicated time-points. Expression values were normalized to the expression of *EIF2C2*, used as standard control. Graph represents the relative expression values of *GOLPH3* messenger RNA (mRNA) relative to cells in G0 (0 h). (**F**) Western analysis of thymidine-nocodazole synchronized U2OS cells showing *GOLPH3* expression after release from mitosis (0 h). *, *p* < 0.05.

The Golgi complex undergoes organelle growth during G1 through S phase of the cell cycle [4]. We reasoned that transcription of Golgi-specific genes may be similarly regulated during the cell cycle, to duplicate all the Golgi components in a timely fashion before cell division. To test this hypothesis, we synchronized U2OS cells in mitosis through a treatment with thymidine followed by nocodazole, as shown in Figure 1A. Cells were subsequently released from mitotic block and cultured in drug-free medium. At several time points, entry and progression of the cells through G1 and into S was confirmed by flow cytometry (Figure 1B), and by assessing the messenger RNA (mRNA) levels of *E2F1* and *CYCE1* (Figure 1C), two cell cycle-regulated genes whose expression is induced in G1 and in S-phase, respectively.

Synchronized RNA samples were used to analyze the expression of several genes coding for Golgi proteins that are known to have structural or enzymatic function (*GOLPH3, GOLGA2, GOLGA5, MAN2A1*). Whereas the expression of *GOLGA5* and *MAN2A2* remained essentially constant through G1, the expression of *GOLPH3* and *GOLGA2* showed a G1-specific induction. Their mRNA levels were increased moderately but significantly about 1.6- to-1.8-fold in early G1 (Figure 1D), suggesting that *GOLPH3* and *GOLGA2* expression is regulated during G1, concomitant with the growth of Golgi organelle.

To confirm cell cycle-dependent regulation of genes encoding Golgi proteins in another cellular system, we made use of mouse NIH 3T3 cells that were synchronized in G0 by serum starvation, and subsequently stimulated with 20% serum to induce entry of the cells into the cell cycle. We focused our analysis on *GOLPH3*, a particularly interesting Golgi peripheral membrane protein involved in secretory trafficking that is oncogenic [3,14,15]. As with U2OS cells synchronized in mitosis by thymidine-nocodazole treatment, the expression of *GOLPH3* mRNA was induced in early G1, and was reduced as cells entered S-phase (Figure 1E). Cell cycle-regulation of *GOLPH3* expression was also observed at the protein level (Figure 1F).

### 3.2. GOLPH3 Gene Promoter Activity Is Regulated by E2F Family Members

**Figure 2:**
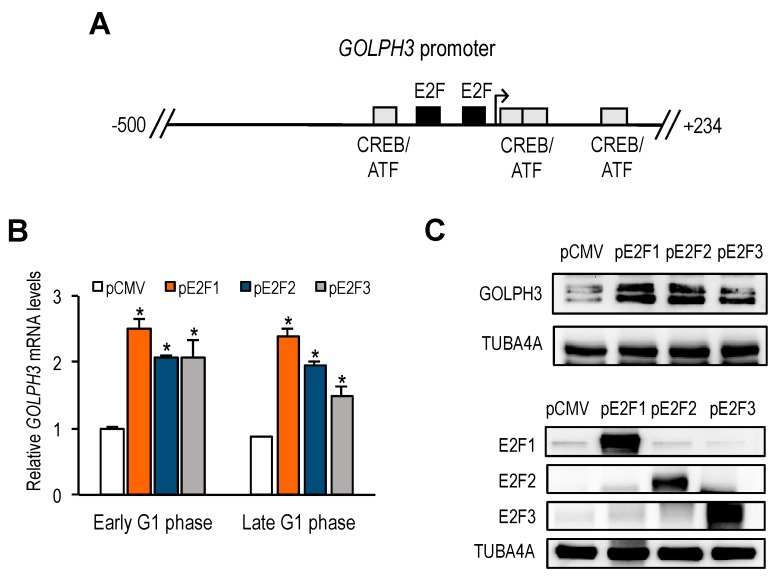
Identification of *GOLPH3* regulatory sequences. (**A**) Schematic representation of transcription factor-binding sites in human *GOLPH3* regulatory region (−500 to +234). E2F and ATF/CREB motifs are indicated as boxes. The transcriptional start site is depicted with an arrow. (**B**) RT-qPCR analysis of *GOLPH3* mRNA expression in U2OS cells transfected with plasmids encoding E2F1, E2F2 or E2F3 proteins under CMV promoter, synchronized with Thy-Noc and subsequently released into the cell cycle. Expression values were normalized to the expression of *EIF2C2*, used as standard control. Data are represented as fold-change (mean ± SD) relative to values of cells transfected with empty pCMV vector. *, *p* < 0.05. (**C**) Western analysis of *GOLPH3* protein levels in U2OS cells overexpressing E2F1-3. Bottom panel shows E2F1, E2F2 and E2F3 protein levels after transfection of each E2F expression plasmid. Shown are the results of one representative experiment of three independent experiments.

To explore the mechanism by which *GOLPH3* is transcriptionally regulated, we examined the sequence of the *GOLPH3* gene promoter to identify transcription factor motifs that could account for its cell cycle-regulated expression. A search for *cis*-regulatory elements in *GOLPH3* genomic sequences with the *ConSite* tool revealed the presence of two E2F binding sites located in the opposite orientation (ACGCCAAA and TCTCCCGCG), shown in Figure 2A. Other Golgi-specific gene promoters also exhibited E2F binding sites in their promoter sequences (Supplementary Figure S1). Furthermore, ENCODE data extracted from Genome Browser database (https://genome.ucsc.edu/cite.html) showed detectable E2F binding activity on their promoters (Supplementary Figure S2), which was strong in the case of *GOLPH3*, *GOLGA5* and *MAN2A1*, and weak in the case of *GOLGA2*, thus correlating with the number of putative E2F sites.

E2F transcription factors are well-known for their role in regulating the timely expression of genes necessary for DNA replication and cell cycle progression [10]. However, their role in Golgi-specific gene expression has not been explored to date. To determine whether *GOLPH3* expression is regulated by E2F factors, we transfected U2OS cells with plasmids encoding E2F1, E2F2 or E2F3, and subsequently synchronized the cells with thymidine-nocodazole. After release from mitotic block, cells were collected in early and late G1-phase (6 h and 9 h). RT-qPCR and Western analyses of the samples showed a significantly increased expression of *GOLPH3* mRNA in cells overexpressing E2F1-3, compared to control cells transfected with empty vector (Figure 2B,C), suggesting that *GOLPH3* is an E2F responsive gene.

**Figure 3:**
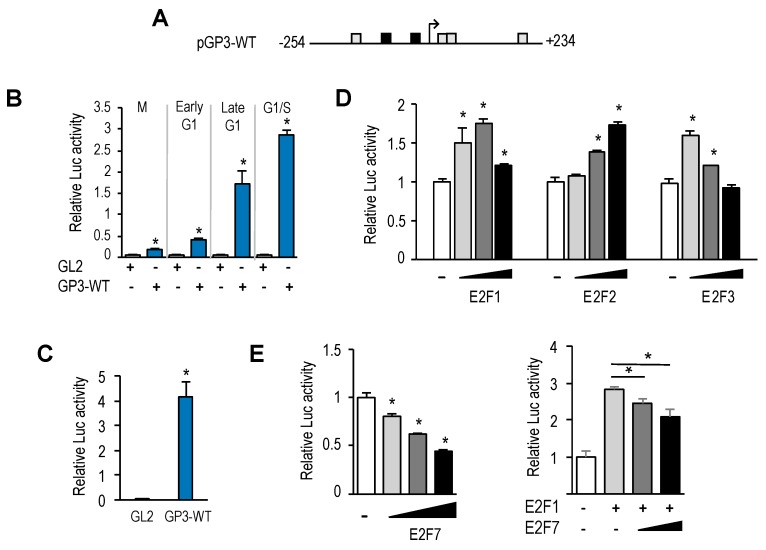
*GOLPH3* gene is regulated by E2F factors at the transcriptional level. (**A**) Promoter-luciferase construct of *GOLPH3*. The nucleotide positions of the promoter construct are numbered relative to the transcription start site. (**B**) Activation of *GOLPH3* promoter-driven firefly luciferase reporter construct during the cell cycle. U2OS cells were transfected with pGP3 wild type construct (pGP3-WT) or pGL2 empty construct along with pRL-TK. Cells were subsequently synchronized in mitosis with Thy-Noc, and released into the cell cycle. Values are represented as firefly luciferase activities relative to Renilla luciferase activities of each sample. (**C**) Activation of *GOLPH3* promoter-driven construct in asynchronous cells. Asynchronously growing U2OS cells were transfected with pGP3-WT construct or pGL2 empty construct along with pRL-TK. Values are represented as firefly luciferase activities relative to Renilla luciferase activities of each sample. (**D**) Ectopic E2F1-3 expression induces *GOLPH3* promoter activity. Asynchronously growing U2OS cells were transfected with pGP3-WT and increasing amounts of E2F1-3 (50 ng, 150 ng, 250 ng) per well in a 12-well plate. Values are represented as luciferase activities relative to pGP3-WT activity of cells transfected with empty pCMV control. (**E**) Ectopic E2F7 expression represses *GOLPH3* promoter activity. Asynchronously growing U2OS cells were transfected with pGP3-WT and increasing amounts of E2F7 (50 ng, 150 ng, 250 ng) in the absence (left panel), or in the presence of E2F1 co-expression (150 ng). Values are represented as luciferase activities relative to pGP3-WT activity of cells transfected with empty pCMV control. Shown are the results of a representative experiment of three independent experiments. *, *p* < 0.05.

To examine transcriptional regulation of the *GOLPH3* gene, we amplified a sequence 5′ to the translational start site of the *GOLPH3* gene (+235) from human genomic DNA using PCR, and cloned the *GOLPH3* regulatory region upstream of a luciferase reporter gene, to generate plasmid pGP3-WT-luc (Figure 3A). This plasmid carries a ≈500 bp regulatory sequence of *GOLPH3* (−254 bp to +234 bp), which includes both E2F sites identified bioinformatically. U2OS cells were transfected with pGP3-WT-luc plasmid or with empty pGL2-luc plasmid, and subsequently synchronized, as detailed above. Luciferase activity was measured at 0 h (mitosis), 4 h, 8 h and 12 h (G1 to S-phase progression). As shown in Figure 3B, luciferase activity was barely detectable in mitotic cells, with values close to those gathered for empty backbone plasmid pGL2. By contrast, luciferase activity increased significantly as cells progressed through the G1-phase of the following cell cycle, suggesting that the *GOLPH3* promoter is responsive to cell cycle regulatory signals. Furthermore, steady-state levels of *GOLPH3* promoter activity were easily detectable in asynchronous cells, most of which are known to be in G1 (Figure 3C).

Given the well-known role of E2F factors as transcriptional regulators, whereby E2F1-3 are thought to activate gene transcription and E2F7 is thought to repress gene transcription [16,17], we expressed E2F1-3 factors individually, along with pGP3-WT-luc in U2OS cells. In all three cases, we found a rise in *GOLPH3* promoter activity upon overexpression of E2F1-3 (Figure 3D). By contrast, when we overexpressed E2F7, alone or in combination with E2F1, the luciferase activity of *GOLPH3* was reduced in a dose-dependent manner (Figure 3E). Taken together, these results suggest that E2F factors are key regulators of *GOLPH3* gene expression at the transcriptional level, and that its regulation is subject to activation and repression, depending on the particular E2F member that may be expressed in cells at a given moment.

### 3.3. Mutational Analysis of GOLPH3 Promoter

**Figure 4:**
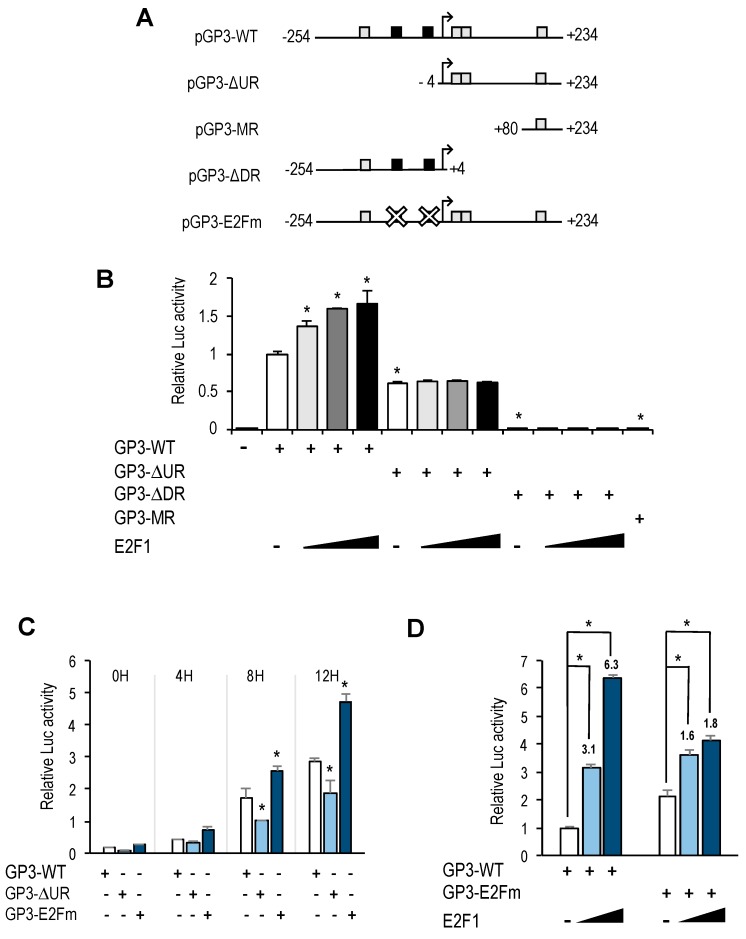
E2F binding motifs function as negative and positive regulatory elements. (**A**) Promoter-luciferase constructs of *GOLPH3* and its derivatives. Mutant motifs are depicted with a white cross. (**B**) Asynchronous U2OS cells were transfected with the indicated luciferase reporter constructs along with pRL-TK and various amounts of a plasmid expressing E2F1 (50 ng, 150 ng, 250 ng) per well in a 12-well plate. Values are represented as luciferase activities relative to pGP3-WT activity of cells transfected with empty pCMV control. (**C**) U2OS cells were transfected with the indicated reporter constructs along with pRL-TK and synchronized in mitosis. Subsequently, cells were released into the cell cycle and luciferase values were calculated as relative values of firefly luciferase activities relative to Renilla activities in each sample. (**D**) Asynchronous U2OS cells were transfected with the indicated reporter constructs and various amounts of a plasmid expressing E2F1 (50 ng, 250 ng). Values are represented as luciferase activities relative to pGP3-WT activity of cells transfected with empty pCMV control. Shown are the results of a representative experiment of two independent experiments. *, *p* < 0.05.

To further analyze *GOLPH3* transcriptional regulation, we generated several promoter deletion constructs (Figure 4A). We first eliminated the 250 bp upstream regulatory region relative to the transcriptional start site, which contained the E2F binding sites, to produce pGP3-ΔUR-luc. This deletion construct, which encompasses the 5′UTR of *GOLPH3*, was transfected into U2OS cells, along with increasing doses of E2F1. Luciferase reporter assays showed that this promoter still maintained a steady-state activity and could be induced during G1-phase, although it was reduced to about half the activity of the pGP3-WT-luc plasmid (Figure 4B,C). Importantly, this construct was unresponsive to E2F1 activity, and luciferase values remained similar to those in samples without E2F1 (Figure 4B). Further deletion of the promoter from −254 to +80 gave rise to plasmid pGP3-MR-luc (Figure 4A). This construct carrying a minimal piece of the *GOLPH3* regulatory region was unable to elicit any luciferase activity under steady-state conditions or upon ectopic E2F1 expression (Figure 4B). Taken together, these results suggest that the upstream region of *GOLPH3* promoter is important for its optimal transcriptional activity, and that E2F factors act through this upstream region.

To specifically determine the role of E2F factors in *GOLPH3* promoter regulation, we constructed a plasmid carrying point mutations in both E2F sites (pGP3-E2Fm-luc), which abrogate E2F binding (Figure 4A and Supplementary Table S2). Interestingly, the activity of the E2F binding site-mutant *GOLPH3* promoter was approximately 1.5 higher than the activity of the wild-type promoter in U2OS cells (Figure 4C,D), suggesting that these E2F sites can function as negative regulatory elements.

We next examined the effect of overexpressing E2F1 in pGP3-E2Fm-luc (Figure 4D). Luciferase analysis showed that E2F1-mediated activation of this reporter plasmid was significantly reduced (from 3-fold and 6-fold induction with the wild-type promoter to 1.6-fold and 1.8-fold with the mutant promoter), suggesting that E2F factors regulate *GOLPH3* gene expression through E2F sites located in its promoter.

### 3.4. A CREB/ATF Site in the GOLPH3 Promoter Regulates Steady-State GOLPH3 Expression

The bioinformatic analysis identified several CREB/ATF motifs in the regulatory region of *GOLPH3*. One motif is located in the region upstream of the transcriptional initiation site, and three more are located downstream of it (Figure 2A). To further investigate the transcriptional regulation of *GOLPH3* gene, we constructed a plasmid lacking the 5′UTR sequence by deleting the region between +5 and +253 (pGP3-ΔDR-luc), as shown in Figure 4A. This plasmid maintains the E2F sites. Surprisingly, we found that basal transcriptional activity of this deletion construct was reduced dramatically, and that E2F expression could not induce it (Figure 4B).

**Figure 5:**
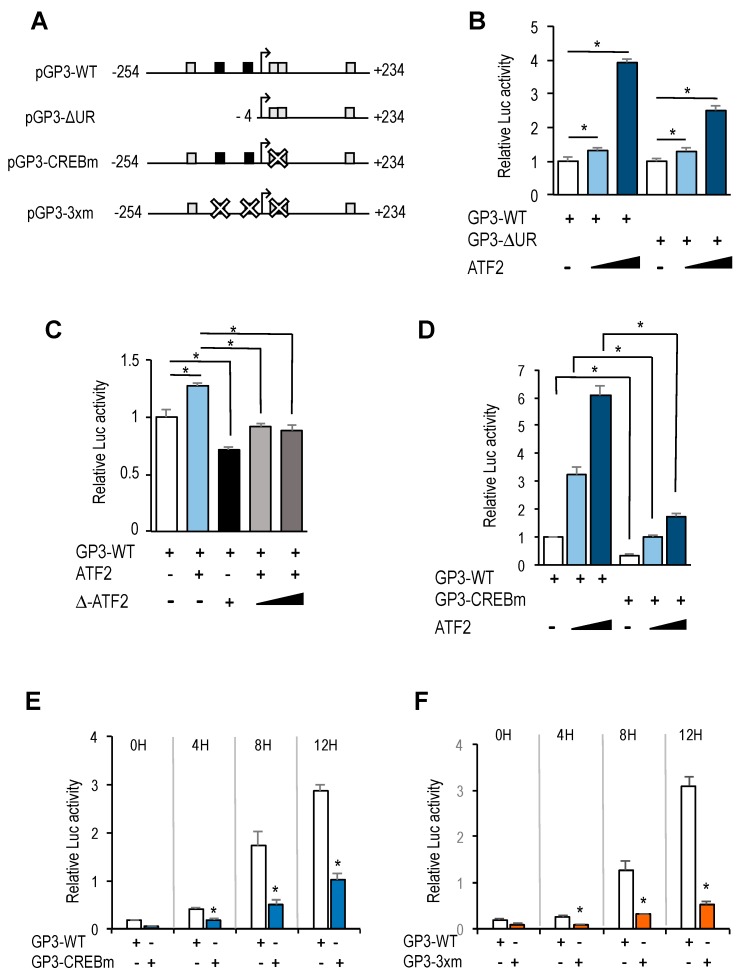
CREB/ATF binding motifs are critical regulators of *GOLPH3* promoter activity. (**A**) Promoter-luciferase constructs of *GOLPH3* and its derivatives. Mutant motifs are depicted with a white cross. (**B**) Asynchronous U2OS cells were transfected with the indicated luciferase reporter constructs along with pRL-TK and various amounts of a plasmid expressing ATF-2 (150 ng, 300 ng). Values are represented as luciferase activities relative to pGP3-WT or pGP3-∆UR activity of cells transfected with empty pCMV control. (**C**) U2OS cells were transfected with pGP3-WT along with a plasmid expressing ATF2 (300 ng), a plasmid expressing Δ-ATF2 (300 ng) or both. Values are represented as luciferase activities relative to pGP3-WT activity of cells transfected with empty pCMV control. (**D**) Asynchronous U2OS cells were transfected with the indicated reporter constructs and various amounts of a plasmid expressing ATF-2 (150 ng, 300 ng). Values are represented as luciferase activities relative to pGP3-WT activity of cells transfected with empty pCMV control. (**E,F**) U2OS cells were transfected with the indicated reporter constructs along with pRL-TK and synchronized in mitosis. Subsequently, cells were released into the cell cycle and luciferase values were calculated as relative values of firefly luciferase activities relative to Renilla activities in each sample. Shown are the results of a representative experiment of two independent experiments. *, *p* < 0.05.

CREB/ATF motifs are frequently regulated by growth promoting signals through the recruitment of members of the CREB/ATF family of transcription factors to these promoter elements [18,19]. We tested whether ATF2 could regulate the activity of the *GOLPH3* promoter. To this end, U2OS cells were transfected with pGP3-WT-luc along with ATF2 or a dominant negative construct of ATF2 (Δ-ATF2) that suppresses both DNA binding and transcriptional activation by wild-type ATF2 [20]. Cells overexpressing ATF2 showed a dose-dependent increase in luciferase activity compared to basal levels (Figure 5B), suggesting that *GOLPH3* expression could be regulated through CREB/ATF sites. Furthermore, the deletion construct carrying the 5′UTR of *GOLPH3* and lacking E2F sites (pGP3-ΔUR-luc) still exhibited ATF2-dependent transcriptional activity (Figure 5B). By contrast, overexpression of Δ-ATF2 did not induce the activity of the *GOLPH3* promoter, and inhibited ATF2-mediated luciferase activity (Figure 5C).

Of the four CREB/ATF sites identified in *GOLPH3* regulatory region, two of them are overlapping (between position +6 and +23), and their score for similarity with the canonical motif is higher than the other two (score of 12 vs. 9, according to values displayed in the *ConSite* analysis). To more directly assess the role of the overlapping CREB/ATF motifs, we mutated both sites (Figure 5A). Transfection of CREB/ATF site mutant in U2OS cells abrogated almost completely the luciferase activity of the *GOLPH3* promoter, and transfection of ATF2 did not increase substantially this activity (Figure 5D). Similarly, luciferase activity was substantially reduced in synchronized U2OS cells transfected with the mutant construct (Figure 5E), and transfection with a construct carrying E2F and CREB/ATF motifs mutated simultaneously resulted in an even further reduced luciferase activity (Figure 5F), suggesting that the E2F and the overlapping CREB/ATF binding motifs are major regulatory sites in cell cycle regulation of *GOLPH3* expression.

### 3.5. GOLPH3 is Required for a Timely G1 Progression

**Figure 6:**
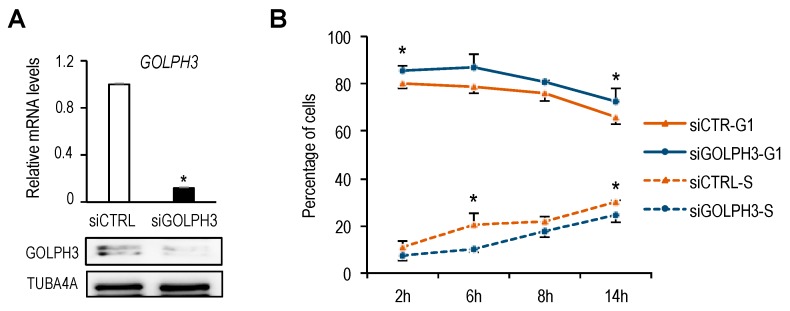
*GOLPH3* expression is necessary for timely G1-phase progression. (**A**) RT-qPCR and Western analysis of U2OS cells transfected with non-target control siRNAs (siCTRL) or with siRNAs specific for *GOLPH3* (si*GOLPH3*). mRNA expression values were normalized to the expression of *EIF2C2*, used as standard control. Data are represented as fold-change (mean + SD) relative to siRNA control. *, *p* < 0.05. *GOLPH3* protein levels were assessed by Western analysis with a specific antibody. (**B**) U2OS cells were transfected with control or *GOLPH3*-specific siRNAs and subsequently synchronized in mitosis by Thy-Noc treatment. After release of cells into the cell cycle, samples were collected at several time-points, incubated with PI and cell cycle phases were assessed by FACS analysis. Data represent the mean ± SD of the fraction of siCTRL or si*GOLPH3*-transfected cells in G1 or S at each time-point obtained from three independent experiments. *, *p* < 0.05.

Golgi organelle growth is thought to be a required step for cells to progress through a “cell growth checkpoint” in late G1 [4]. Given our finding of a timely induction of *GOLPH3* expression during G1, we wondered whether changes in *GOLPH3* levels would impact cell cycle progression. To analyze this possibility, we transfected U2OS cells with siRNA molecules specific for *GOLPH3* [12] in order to silence *GOLPH3* mRNA expression (Figure 6A). Cells were subsequently synchronized with thymidine-nocodazole and cell cycle progression was examined by flow cytometry. We consistently found that depletion of *GOLPH3* levels results in an increased percentage of cells in G1 and a lower percentage of cells in S-phase, suggesting that reduction in *GOLPH3* levels delays G1-to-S progression (Figure 6B). Thus, *GOLPH3* emerges as a Golgi constituent protein required for timely cell cycle progression.

## 4. Discussion

During cell division, the Golgi organelle must be accurately segregated into the two daughter cells. This segregation is achieved through disassembly of the Golgi cisternae when cells enter mitosis followed by a reassembly of the fragments within each daughter cell after the end of mitosis, through a process involving a wide array of mediators, including Golgi and cytoskeletal proteins, membrane tethers, as well as kinases and phosphatases to transmit signals into the Golgi membranes [21]. Biogenesis of the Golgi apparatus during interphase is less well-studied. There is evidence that the mammalian Golgi apparatus grows in its protein content and volume throughout the G1 phase in a manner that correlates well with cell size growth [4]. However, it is unknown whether signals emanating from the cell cycle regulatory network are conveyed to the Golgi organelle for its regulated growth during G1. Here we show that the expression of *GOLPH3* and *GOLGA2*, two genes encoding Golgi proteins, is induced specifically in the G1 phase, suggesting a link between cell cycle regulation and Golgi growth. Furthermore, we present evidence that E2F transcription factors, critical regulators of the G1 to S transition of the cell cycle regulate expression of *GOLPH3*. Thus, E2F factors may coordinate not only DNA replication, but also Golgi-specific gene expression to contribute to the timely cell cycle progression.

The genes selected for our initial gene expression analysis (*GOLPH3*, *GOLGA2*, *GOLGA5* and *MAN2A1*) are well-known for their Golgi-specific functions [22]. These genes bear putative E2F sites in their promoter regions and show detectable E2F binding activity on their promoters. The presence of E2F sites raised the possibility that these genes that encode Golgi proteins may be regulated in the cell cycle. However, we found differences in their expression pattern during the G1 to S phase. *GOLPH3* and *GOLGA2* clearly exhibit cell cycle regulated expression at the mRNA level. By contrast, *GOLGA5* and *MAN2A1* did not show such regulation, suggesting that the E2F elements present in their promoters are not functional in our experimental system. A more systematic analysis involving several cell lines and various cell cycle synchronization conditions is needed to determine to what extent Golgi-specific genes are transcriptionally regulated in the cell cycle. Alternatively, Golgi components may be regulated at the protein level. There is evidence of posttranscriptional mechanisms regulating the expression of some Golgi proteins. For example, *GOLGA2* and *MAN2A1* levels have been shown to be induced at the translational level during interphase in an S6K1-dependent manner [4]. Thus, Golgi protein levels appear to be regulated by a combination of cell cycle-regulated transcriptional and posttranscriptional mechanisms that would ensure the necessary increase of Golgi size before cell division.

*GOLPH3* promoter harbors two putative E2F motifs, and our promoter deletion and mutation analyses indicate that regulation of *GOLPH3* expression by E2Fs is mediated through these E2F sites. A comparison of *GOLPH3* E2F motifs between several mammalian species shows that the proximal E2F site is more conserved than the distal E2F site (Supplementary Figure S2), suggesting that both E2F sites may not be functionally equivalent. Mouse and rat *GOLPH3* genes only harbor the E2F site that corresponds to the proximal site in humans, and our data show that mouse *GOLPH3* expression is induced well in G1 phase, similarly to human *GOLPH3* gene, which suggests that the proximal E2F motif may be more functionally active than the distal E2F motif in the human promoter. A mutational analysis of individual E2F sites may help define more precisely the contribution of each motif to the regulation of *GOLPH3* gene expression.

Similarly to many other E2F-regulated genes, *GOLPH3* expression is induced by E2F1-3 and repressed by E2F7. Gene expression during G1 to S progression is thought to be mediated by a complex set of regulatory feedback loops established among members of the E2F family [23]. According to this model, induction of G1 genes by E2F1-3 would subsequently be repressed by the activity of the repressor arm of the E2F family (E2F6-8), whose own expression follows temporally that of E2F1-3. Our results suggest that the coordinated action of E2F activators and E2F repressors would ensure a tightly regulated expression of *GOLPH3*, and perhaps other genes encoding Golgi proteins, within the G1 to S window, prior to Golgi dispersal during mitosis. Thus, E2F factors may be major determinants of Golgi-specific *GOLPH3* gene regulation.

Interestingly, the E2F sites themselves function as negative regulatory elements, as shown by the fact that mutation of E2F motifs leads to an increased basal promoter activity (Figure 4B,C). A similar type of regulation has been reported for several other E2F target genes, including *MYB*, *E2F1*, *BRCA1*, and it is thought to result from recruitment of E2F repressor complexes containing Retinoblastoma family members [24,25,26]. In this context, *GOLPH3* expression would be regulated negatively in G0 or early G1 by RB-dependent repression and in S-phase by E2F7-dependent expression, with a window in G1 that would allow its induction by E2F1-3.

In addition to E2F motifs, our luciferase reporter data indicate that other regulatory elements in *GOLPH3* promoter have a critical role in *GOLPH3* expression. Most notably, we have identified four CREB/ATF binding motifs that are located in its regulatory region. Two overlapping motifs located adjacent to the transcriptional start site are well conserved in other mammalian genomes, whereas the other two show greater divergence (Supplementary Figure S3). Mutation of both CREB/ATF overlapping motifs reduces dramatically steady-state activity of *GOLPH3* promoter, suggesting that these elements are essential for *GOLPH3* expression.

The CREB/ATF motif is recognized by proteins of the CREB and ATF families [18]. Members of these families are structurally similar, but have discrete transcriptional properties that contribute to their distinct regulatory functions [27]. We presently do not know which individual members are recruited to CREB/ATF motifs in *GOLPH3* promoter to regulate its expression. We find that ectopic expression of ATF2, a transcription factor that can be activated by growth promoting signals, is able to induce *GOLPH3* promoter activity, suggesting that activation of ATF2 early in G1 could contribute to the induction of *GOLPH3* transcription through its CREB/ATF elements. Nevertheless, individual silencing of CREB and ATF family members may give a more comprehensive picture of *GOLPH3* regulation by these transcription factors, known to be activators or repressors depending on the cellular context. Of note, some CREB/ATF family members partially localize to the Golgi [28]. It will be interesting to analyze their role in the regulation of *GOLPH3* transcription.

It has been suggested that Golgi growth is modulated by a “cell growth checkpoint” at late G1 phase, whereby cell growth signals delivered by S6K1 would control the accumulation of some Golgi proteins and therefore Golgi size [4]. Our data suggests an additional layer of regulatory mechanism for Golgi growth by identifying a transcriptional mechanism regulating *GOLPH3* expression in G1. Importantly, the delay in G1 to S progression after *GOLPH3* depletion that we have observed suggests the existence of a “Golgi growth checkpoint”, which may impair cell cycle progression if cells are expressing suboptimal amounts of Golgi proteins. Further research is needed to define the mechanism for this regulation.

## Supplementary Materials

The following are available online at https://www.mdpi.com/2073-4425/10/3/247/s1, Figure S1: Schematic representation of promoters of genes encoding Golgi proteins. Figure S2: Graphical representation of E2F transcription factor binding to the promoter regions of indicated genes. Figure S3: Sequence alignment of the E2F and CREB/ATF regulatory elements in *GOLPH3* promoter across species. Table S1: Nucleotide sequences of primers used for construction of deletion mutants of *GOLPH3* promoter. Table S2: Nucleotide sequences of primers used for construction of E2F and CREB/ATF motif mutants of *GOLPH3* promoter. Table S3: Nucleotide sequences of primers used for RT-qPCR experiments.
