# Supplementary material for: Golgi Oncoprotein GOLPH3 Gene Expression Is Regulated by Functional E2F and CREB/ATF Promoter Elements
Source: Genes (Basel). 2019 Mar 25;10(3):247. doi: 10.3390/genes10030247 (PMC6471639; doi:10.3390/genes10030247)
Supplement: Supplementary file 1 [file genes-10-00247-s001.pdf]

Supplementary information

To accompany Peñalver-González, Vallejo-Rodríguez et al., "Golgi Oncoprotein *GOLPH3* Gene Expression is Regulated by Functional E2F and CREB/ATF Promoter Elements "

Figure Legends

Supplementary Figure 1. Schematic representation of promoters of genes encoding Golgi proteins. E2F and CREB/ATF transcription factor-binding sites in human *GOLGA2, GOLGA5 and MAN2A1* regulatory regions (-500 to +234) are indicated as boxes. The transcriptional start site is depicted with an arrow.

Supplementary Figure 2. Graphical representation of E2F transcription factor binding to the promoter regions of indicated genes. H3K27Ac enhancer marks are also included for each gene.

Supplementary Figure 3. Sequence alignment of the E2F and CREB/ATF regulatory elements in *GOLPH3* promoter across species. Sequences of the human, chimpanzee, mouse, rat and cow *GOLPH3* promoters are depicted. The asterisk denotes an identical nucleotide in all species tested. Lowercase letter denotes a non-transcribed sequence; uppercase letter denotes a transcribed sequence.

SUPPLEMENTARY FIGURE 1

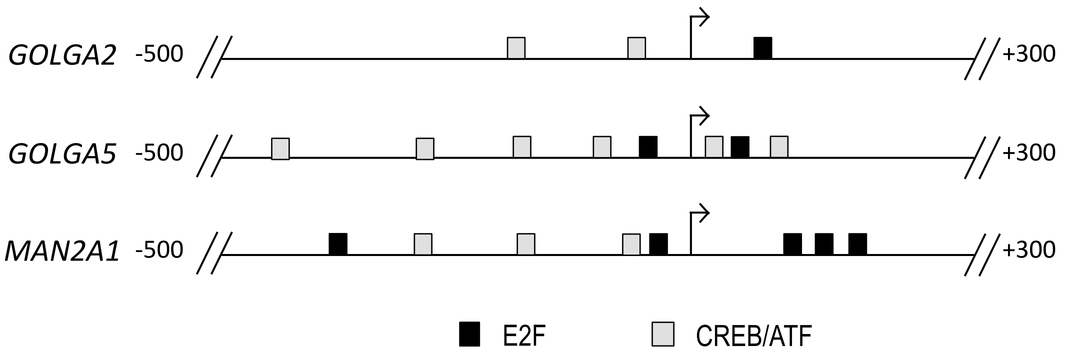

SUPPLEMENTARY FIGURE 2

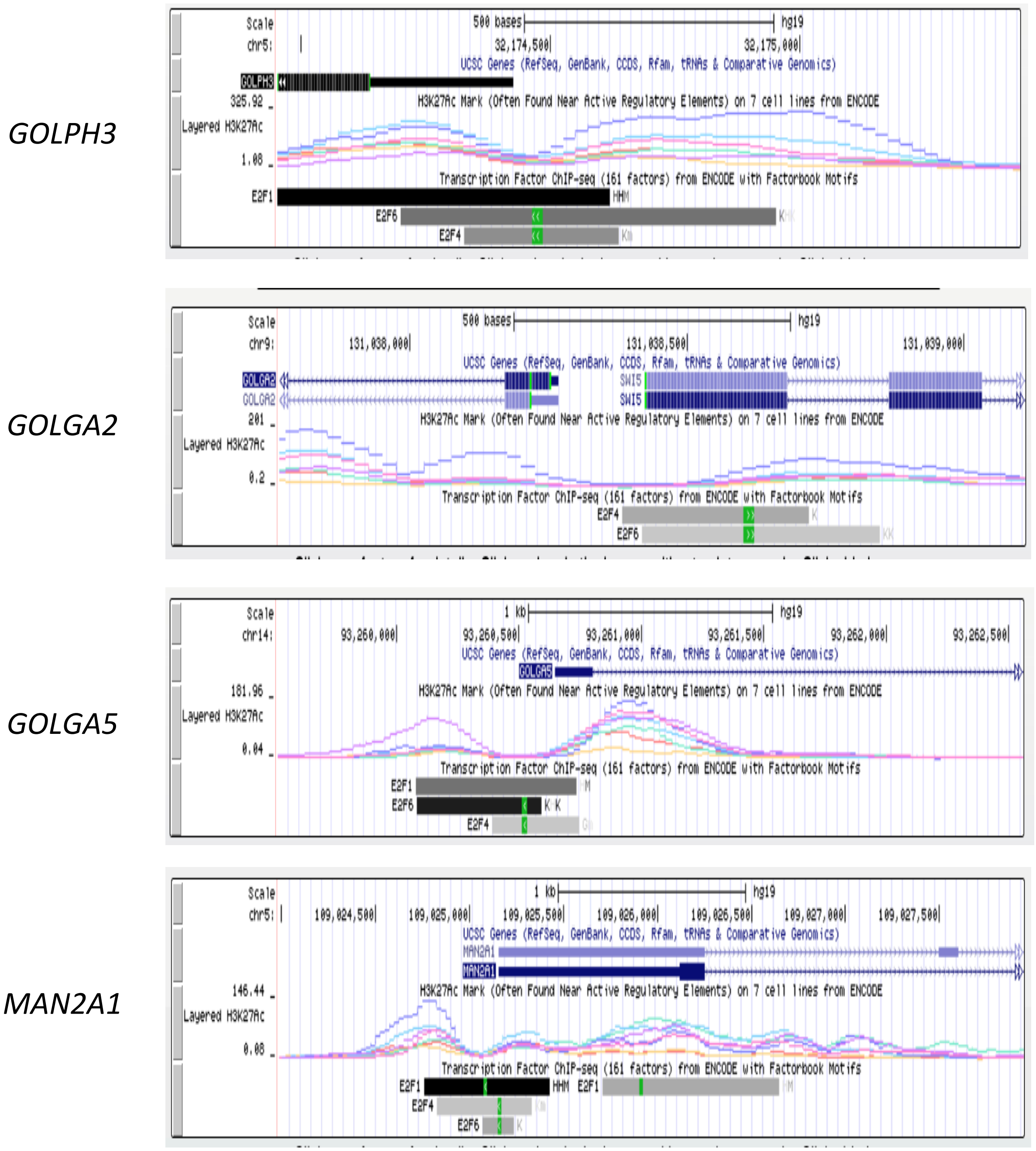

SUPPLEMENTARY FIGURE 3

```
                      CREB/ATF       E2F        E2F          CREB/ATF        CREB/ATF
GOLPH3_Human         cacgtcacggga  acgccaaa  ctcccgcg  TCCGGTGACGTCAGCCGG  GACTGCCCCCG
Golph3_Chimpazee     CACGTCACGGGA  ACGCCAAA  CTCCCGCG  TCCGGTGACGTCAGCCGG  GACTGCCCCCG
Golph3_Mouse         gaactgagaggc  --------  gccccgcg  tccggtgacgtcagcgcg  GACCGCCTTCG
Golph3_Rat           gaactgtgaggc  --------  gccccgcg  tccggtgacgtcaacgcg  gactgccttcg
Golph3_Cow           cacgtcacggga  actctaag  gccccgcg  tccagtgacgtcagccgg  GACTGCATCCA
                      *  *    **   ** * **     ******  *** ********* *  *  ******   *
```

Supplementary Table S1: Nucleotide sequences of primers used for construction of deletion mutants of *GOLPH3* promoter

Primer	Sequence	R.E.* added	Size (bp)
pGL2-G3P-WT_F	TGC AGA CGC GTC CCA GGC TCT TCC ATT CAC	MluI	488
pGL2-G3P-WT_R	TGC AGA AGC TTR CCG GGT TTC CGT GTT AAA T	HindIII	
pGL2-G3P-ΔUR_F	TGC AGA CGC GTC CCA GGC TCT TCC ATT CAC	MluI	281
pGL2-G3P-ΔUR_R	TGC AGA AGC TTG ATG TGG CAG AGT CG	HindIII	
pGL2-G3P-ΔDR_F	TGC AGA CGC GTT GCC ACA TCC TCC GGT GAC	MluI	260
pGL2-G3P-ΔDR_R	TGC AGA AGC TTT CCG GGT TTC CGT GTT	HindIII	
pGL2-G3P-MR_F	TGC AGA CGC GTC GGG GAC TGC GGC CAC	MluI	176
pGL2-G3P-MR_R	TGC AGA AGC TTT CCG GGT TTC CGT GTT	HindIII	

* R.E., restriction endonuclease

Supplementary Table S2: Nucleotide sequences of primers used for construction of E2F and CREB/ATF motif mutants of *GOLPH3* promoter

Primer	Sequence
pGL2-G3P-E2F Mutants (E2F Mut1, E2F Mut2, E2F Mut1-2)_F1	GGG GGC GCA GGA cCa CtA gAG AAA AGA CAA GCA CCG AGG
pGL2-G3P-E2F Mutants (E2F Mut1, E2F Mut2, E2F Mut1-2)_F2	GCC TCC CGG CCC AGC TCg Cat GtG aCG ACT CTG CCA CAT
pGL2-G3P-CREB Mut_F	TCC TCC GGT cca tgC AGC CGG GGC CGC CAT ATT GGA AAG GCG
pGL2-G3P-CREB Mut_R	GTT GCC ACA TCC TCC GGT cca tgC AGC CGG GGC CGC CAT

Supplementary Table S3: Nucleotide sequences of primers used for RT-QPCR experiments

Primer	Sequence
qH_GOLPH3_F	CTG GAT TAC GTG GCT GTA TGT
qH_GOLPH3_R	CCT GTT GGA GCA TCT GAC TT
qH_GOLGA2_F	ACG GAT CAG TTG GAA GAA GAA A
qH_GOLGA2_R	GGA TCC CTA TGG TCT GAA TGT G
qH_MAN2A1_F	TGG TGC CTC ATT CCC ATA AC
qH_MAN2A1_R	CTT CCT CCG TGA GTC TTC TTT C
qH_GOLGA5_F	GCA GAG GGA GGA AAT ACA GAA G
qH_GOLGA5_R	ACT TGC TGT GCC TCC ATA TC
qH_EIF2C2_F	GTC CCT TTT GAG ACG ATC CAG
qH_EIF2C2_R	AGC CAA ACC ACA CTT CTC G
